# Basal Ganglia Calcifications for Nephrologists: Fahr/Primary Familial Brain Calcification (PFBC) at the Interface of Chronic Kidney Disease-Mineral and Bone Disorder (CKD-MBD) and Hypoparathyroidism

**DOI:** 10.7759/cureus.99031

**Published:** 2025-12-12

**Authors:** Fahad S Alrashidi

**Affiliations:** 1 Internal Medicine/Nephrology, Ad Diriyah Hospital/Riyadh Third Health Cluster, Riyadh, SAU

**Keywords:** basal ganglia calcification, computed tomography, fahr syndrome, hypoparathyroidism, movement disorder, neurovascular unit, primary familial brain calcification, seizure

## Abstract

Bilateral basal ganglia calcifications are increasingly recognized on neuroimaging, yet their interface with chronic kidney disease-mineral and bone disorder (CKD-MBD) and hypoparathyroidism is rarely addressed from a nephrology perspective. In this narrative review, we synthesized data on primary familial brain calcification (PFBC) and secondary Fahr syndromes, highlight proposed mechanisms linking disturbances in calcium-phosphate-parathyroid hormone (PTH) homeostasis to intracranial parenchymal calcification, and summarize the clinical spectrum from incidental findings to disabling movement, cognitive, and psychiatric manifestations. Targeted searches of the literature and relevant guidelines were used to integrate mechanistic, imaging, and clinical studies with the authors’ nephrology experience. Evidence suggests that chronic hypocalcemia with relative hyperphosphatemia in hypoparathyroidism and disordered calcium-phosphate-PTH balance in CKD-MBD (typically secondary hyperparathyroidism) may lower the threshold for basal ganglia mineralization. Symptom control and early metabolic correction can improve neurological outcomes even though radiologic calcifications usually persist. Direct data in dialysis and transplant cohorts remain sparse, representing a major research gap. We propose a pragmatic, nephrology-focused approach: confirm the radiologic pattern, obtain a compact mineral panel in all patients, treat reversible metabolic causes before extensive testing, and reserve PFBC genetic panels for compatible phenotypes and family histories. This framework aims to make basal ganglia calcifications a more familiar and actionable finding in kidney practice.

## Introduction and background

Bilateral, largely symmetric calcifications within the basal ganglia (most commonly the globus pallidus and putamen) and the cerebellar dentate nuclei have fascinated clinicians and pathologists for nearly a century. The label “Fahr” was historically applied to idiopathic and familial entities and, at times, loosely to any conspicuous calcification of deep gray matter. Current usage is more disciplined: the term primary familial brain calcification (PFBC) denotes the genetic/idiopathic group, whereas “Fahr syndrome” is often reserved for secondary causes-most prominently disorders of the calcium-phosphate-parathyroid hormone (PTH) axis. Despite the semantic nuance, the practical message is simple: symmetrical calcifications on computed tomography (CT) are a radiologic pattern, not a diagnosis. Etiological assignment requires clinical context, basic laboratory testing, and, when indicated, genetic evaluation. Because secondary forms are potentially reversible at the level of symptoms (even if calcifications persist), timely recognition can improve function and quality of life.

## Review

Methods

This work was conducted as a narrative literature review rather than a formal systematic review. We performed targeted searches of PubMed/MEDLINE, Embase, and Google Scholar using combinations of the following keywords and MeSH terms: "basal ganglia calcification", "Fahr syndrome", "primary familial brain calcification", "PFBC", "intracranial calcification", "hypoparathyroidism", "chronic kidney disease", "CKD-mineral and bone disorder", "dialysis", and "transplant". Searches were initially performed in January 2024 and updated through October 2025 to capture recent publications. We prioritized human studies published in English that addressed one or more of the following domains: (1) clinical and radiologic features of basal ganglia calcifications (BGC), (2) genetic forms (PFBC) and their proposed mechanisms, (3) hypoparathyroidism-related or other secondary Fahr syndromes, and (4) links between chronic kidney disease-mineral and bone disorder (CKD-MBD), dialysis or transplantation, and intracranial calcification. Case reports, case series, observational cohorts, mechanistic and imaging studies, and relevant guideline or consensus documents were all eligible. Reference lists from key articles and reviews were hand-searched to identify additional pertinent reports, including those from Saudi Arabia and the wider Middle East. Because this was a narrative review, we did not apply a predefined Preferred Reporting Items for Systematic Reviews and Meta-Analyses (PRISMA)-style protocol, perform a formal risk-of-bias assessment, or undertake quantitative meta-analysis. Instead, the aim was to synthesize the available evidence, highlight areas of agreement and uncertainty, and integrate the literature with the authors’ clinical perspective as nephrologists caring for patients with CKD-MBD and suspected Fahr/PFBC.

Historical notes and evolving concepts

Historically, deep gray matter calcifications were described in isolated case reports long before modern neuroimaging. Autopsy studies demonstrated laminated mineral deposits around capillaries and in perivascular spaces, with variable astroglial and microvascular changes. The advent of CT transformed the field by enabling in vivo detection of even minute calcific foci. In contemporary practice, detection is often incidental during work-up of headache, trauma, or non-specific neurological complaints, prompting questions about clinical relevance and appropriate work-up.

Epidemiology

The epidemiology of intracranial calcification is best considered along two axes: (i) incidental radiological findings in unselected CT cohorts and (ii) clinically manifest PFBC or secondary Fahr syndrome. Incidental BGC are reported in a small fraction of CT scans, with prevalence increasing with age and vascular comorbidity. By contrast, the clinical syndromes are rare. The gap between radiologic prevalence and clinical disease underscores that calcification is a biomarker that must be interpreted in context rather than a disease state per se. Importantly, radiologic severity does not perfectly correlate with symptom burden: individuals with striking calcification may be asymptomatic, while some with modest radiologic change present with disabling movement or neuropsychiatric features.

Genetics and pathophysiology

Pathophysiologically, mineral deposition in Fahr and related syndromes likely reflects the interaction between systemic mineral imbalance and local neurovascular vulnerability. Sustained hypocalcemia with relative hyperphosphatemia, as in chronic hypoparathyroidism, increases the calcium-phosphate product and favors precipitation along small-vessel walls and in perivascular spaces of metabolically active deep gray structures. Histopathological descriptions in hypoparathyroid Fahr cases show concentric mineral deposition around arterioles, venules, and capillaries, often accompanied by gliosis, supporting a dynamic process rather than passive “stone formation.” Clinically, acute hypocalcemia generates neuromuscular irritability (tetany, paresthesias, and seizures), whereas chronic, partially treated disease is more often associated with movement disorders and cognitive or psychiatric symptoms. Case series consistently report substantial improvement in seizures, tetany, and encephalopathic features after the normalization of calcium and phosphate, while CT calcifications rarely regress, underscoring that the principal therapeutic target is symptom and function rather than radiologic clearance.

Pathophysiology: from transport to mineralization

Pathophysiologically, mineral deposition likely reflects the convergence of several processes: dysregulated phosphate transport, low-grade microvascular injury with blood-brain barrier leakage, and astrocytic responses that favor ectopic mineralization. Histology has shown concentric, lamellar deposits within perivascular spaces and capillary walls, sometimes admixed with iron and surrounded by astrocytosis or microglial activation. Why certain regions calcify preferentially is unresolved; metabolic demand, regional vascular architecture, and transporter expression may all contribute. Secondary forms linked to hypoparathyroidism provide a natural experiment: sustained hypocalcemia with relative hyperphosphatemia appears to lower the threshold for ectopic brain mineralization, especially in metabolically active deep gray structures.

Population imaging studies suggest that the apparent prevalence of BGC depends strongly on scanner resolution, age structure of the cohort, and ascertainment bias. Community cohorts that deliberately excluded known metabolic disease still identified calcification in older adults, supporting the concept of “physiologic” intracranial calcification that should not automatically trigger extensive work-ups. In contrast, hospital series enriched for metabolic disease or epilepsy report higher rates. Importantly, the anatomical distribution and symmetry remain key: confluent, bilateral involvement of the globus pallidus with extension to dentate nuclei is more suggestive of PFBC/Fahr than scattered cortical or vascular wall calcifications [1-5].

Ethnic or geographic differences are difficult to establish because most series are small. Nonetheless, increasing availability of CT in emergency settings in the Middle East will likely uncover more incidental cases; whether this translates into clinically manifest disease is uncertain. Establishing minimum data sets in radiology reports and cross-referral pathways to neurology/endocrinology could reduce unwarranted variation in downstream testing.

From a nephrology standpoint, PFBC genetics matter for at least three reasons. First, recognizing a familial PFBC pattern prevents over-attribution of calcifications to CKD-MBD alone and prompts appropriate counseling of relatives, including potential living kidney donors. Second, CKD-related disturbances in phosphate balance may accelerate phenotypic expression in PFBC carriers, making optimization of CKD-MBD targets particularly relevant in this subgroup even though direct data are lacking. Third, differentiating genetic PFBC from secondary, hypocalcemic Fahr syndromes shapes follow-up: in PFBC, emphasis is on long-term movement, cognitive, and psychiatric surveillance, whereas in secondary forms the priority is durable correction of the mineral axis.

From a mechanistic perspective, phosphate disequilibrium may act as a final common pathway across genotypes. Loss-of-function variants in SLC20A2 reduce astrocytic uptake of inorganic phosphate, whereas XPR1 variants impair cellular phosphate export; either disturbance may favor local supersaturation and mineral nucleation. PDGFRB/PDGFB variants, by compromising pericyte signaling and blood-brain barrier integrity, could permit leakage of serum proteins that serve as a scaffold for mineral deposition. The recessive genes emphasize that astrocytes (MYORG) and endothelium (JAM2) are not bystanders but active participants in the calcification niche [5-9].

Penetrance is age-dependent: longitudinal family studies show that many obligate carriers are asymptomatic in early adulthood but develop radiologic changes later. This has implications for counseling; clinicians should avoid deterministic language and emphasize modifiable contributors such as vascular risk factor control and avoidance of neurotoxins.

Clinical spectrum

The clinical spectrum spans asymptomatic individuals to patients with prominent movement disorders (parkinsonism, dystonia, chorea, and tremor), gait ataxia due to dentate involvement, neuropsychiatric syndromes (depression, apathy, and psychosis), cognitive impairment, headaches, and seizures. Presentations may be acute (e.g., hypocalcemic tetany with carpopedal spasm and positive Trousseau/Chvostek signs) or insidious (progressive gait or cognitive change). Pediatric cases, while uncommon, often raise differential diagnoses of congenital infection and metabolic disease; careful pattern recognition and laboratory testing are crucial to avoid misattribution. In secondary Fahr syndrome, neurological symptoms frequently improve with metabolic correction; however, the radiological burden of calcification usually remains unchanged, supporting the principle that clinical management should be driven by symptoms and metabolic control rather than serial attempts to “reverse” calcification.

Neuropsychiatric presentations are under-recognized. Depression, irritability, and executive dysfunction may precede motor findings by years. When calcifications are present, clinicians should resist simplistic attributions-comorbid primary psychiatric illness is common in the general population and requires standard care. Conversely, in PFBC families, new-onset psychosis or mood disorders warrant careful neurological assessment, particularly if accompanied by subtle motor signs [10-13].

Peripheral paresthesias and muscle cramps are common manifestations of acute hypocalcemia and may accompany or precede central neurological features. A focused history and examination for perioral or digital numbness, muscle cramps, carpopedal spasm, and other signs of neuromuscular irritability (e.g., Chvostek or Trousseau signs), together with prompt biochemical confirmation, help distinguish hypocalcemic seizures or encephalopathy from alternative causes.

Diagnosis and work-up

A pragmatic diagnostic pathway begins with confirming the radiologic pattern on non-contrast CT, which remains the most sensitive modality for detecting fine parenchymal calcification. MRI can complement CT by excluding alternative pathologies (e.g., neoplasm and demyelination) but is less sensitive to subtle calcific density. Once calcification is documented, a compact metabolic screen should follow in all patients: albumin-corrected serum calcium, phosphate, magnesium, PTH, and 25-hydroxyvitamin D (25(OH)D). In CKD, PTH should be interpreted in context rather than “normalized” to a fixed target; the key diagnostic signal is whether PTH is inappropriately low or inappropriately normal relative to the accompanying calcium/phosphate pattern (suggesting hypoparathyroidism, pseudohypoparathyroidism, or iatrogenic over-suppression/adynamic bone tendency), versus markedly elevated PTH with phosphate retention suggesting secondary hyperparathyroidism. For the purpose of evaluating BGC, the immediate clinical priority is correction of symptomatic hypocalcemia, hyperphosphatemia, and hypomagnesemia and avoidance of iatrogenic extremes of PTH, rather than pursuit of a specific PTH numeric endpoint. Thyroid function, renal indices, and an infectious/toxic screen are ordered selectively according to history and examination. Genetic testing is reserved for patients with typical bilateral, symmetric calcification in whom secondary causes have been excluded, those with a strong family history, or unusually young patients. A tiered approach using a PFBC panel (including SLC20A2, PDGFRB, PDGFB, XPR1, MYORG, JAM2, and CMPK2) is efficient; exome sequencing can be considered if panel testing is unrevealing in a high-suspicion context. Counseling should emphasize incomplete penetrance and variable expressivity: the presence of calcification does not mandate symptoms, and surveillance strategies should be individualized.

A practical algorithm is the following: confirm calcification on non-contrast CT → screen Ca/Phos/Mg/PTH/25(OH)D in all patients → if abnormal, treat and reassess symptoms; if normal and patient is young or has a family history or typical distribution, proceed to PFBC gene panel. Additional tests are guided by context (e.g., HIV or TORCH serology when congenital infection is suspected, toxic exposure history, and autoimmune screen when encephalitic features are present).

A stepwise approach that addresses reversible metabolic abnormalities before advanced testing is resource-conscious and clinically meaningful. In hypoparathyroidism, conventional therapy with oral calcium and active vitamin D (e.g., calcitriol) is recommended to relieve symptoms of hypocalcemia and restore biochemical stability. Accordingly, symptomatic improvement after initiating or optimizing this therapy may support a causal link in patients with BGC, functioning as a pragmatic “therapeutic trial” embedded within standard care [14].

Differential diagnosis and when to suspect PFBC

A working split into “secondary/metabolic” versus “primary genetic (PFBC)” is clinically useful. Secondary causes include hypoparathyroidism (postsurgical, autoimmune, and infiltrative), pseudohypoparathyroidism, chronic hypocalcemia/hyperphosphatemia of CKD, intoxications (e.g., carbon monoxide), infections, and rare mitochondrial or metabolic disorders. PFBC is suspected with a compatible CT pattern, family history, and neurologic syndromes without metabolic triggers. Genetic testing is most informative when (1) calcium-phosphate-PTH-vitamin D panel is normal or only minimally perturbed, (2) there is a clear autosomal-dominant pedigree, or (3) the onset is early or complex despite biochemical control.

A pragmatic diagnostic approach in nephrology settings begins with confirmation of the radiologic pattern on non-contrast CT, ensuring that the calcifications are bilateral and symmetric within the basal ganglia, with or without involvement of the dentate nuclei. Once this pattern is established, all patients should undergo a first-line biochemical panel that includes corrected serum calcium, phosphate, magnesium, alkaline phosphatase, PTH, and 25(OH)D, with thyroid function tests and renal indices added where appropriate. The results of this initial work-up usually allow clinicians to classify patients into those with a likely metabolic or secondary cause-such as hypoparathyroidism and pseudohypoparathyroidism. Patients can be broadly stratified into those with likely secondary, potentially reversible causes of BGC-particularly hypoparathyroidism, pseudohypoparathyroidism, and postsurgical states-and those in whom PFBC is more plausible because the mineral axis is normal or only minimally disturbed.

CKD-related disturbances of the calcium-phosphate-PTH axis (CKD-MBD) may represent an additional but uncommon secondary context, with the strongest support coming from case-based observations, especially in severe or uncontrolled secondary hyperparathyroidism and phosphate retention. In the latter group with a near-normal mineral profile, especially when there is a suggestive family history or an early, complex neurological phenotype, targeted PFBC gene panel testing can be considered. Ancillary investigations should then be tailored to the clinical presentation-for example, electroencephalography when seizures are prominent, or formal neuropsychological assessment when cognitive or psychiatric symptoms predominate.

Imaging hallmarks and reporting

**Figure 1:**
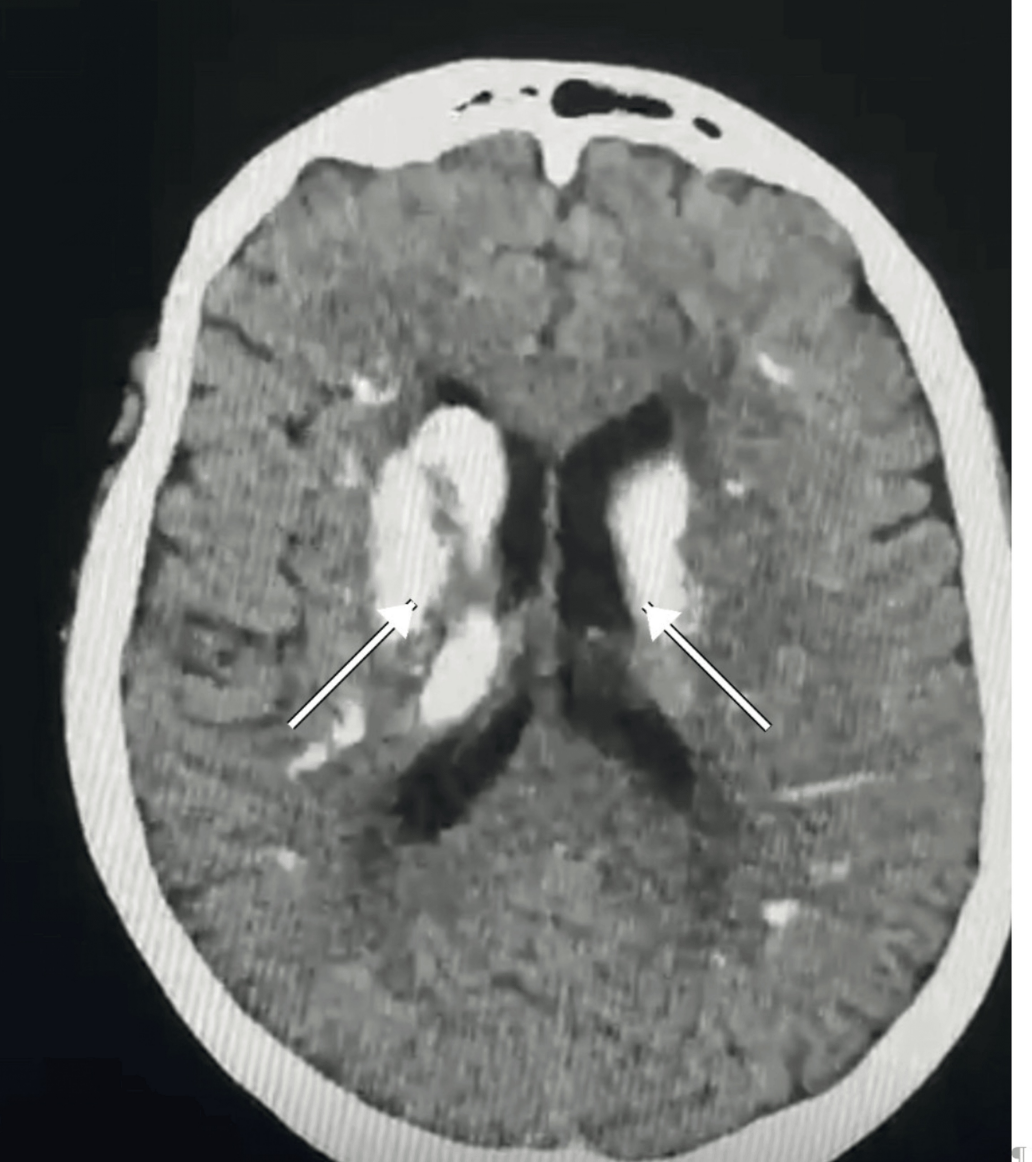
Axial non-contrast CT demonstrating bilateral, symmetric calcifications centered on the globus pallidus (arrows), consistent with Fahr syndrome secondary to hypoparathyroidism CT: computed tomography The figure is an original, de-identified CT image from the author’s clinical practice and has not been previously published.

On CT, calcifications appear as dense, hyperattenuating foci within the globus pallidus and putamen, commonly extending to the dentate nuclei; thalamic and subcortical white matter involvement is variable (see Figure 1). A semi-quantitative approach is useful in reports-documenting laterality (usually symmetric), anatomic distribution, and an estimate of burden (mild/moderate/severe). Differential considerations on imaging include congenital infections (often asymmetric with cortical and periventricular changes), Sturge-Weber syndrome (tram-track cortical calcifications), prior radiotherapy, and calcified neoplasms. Correlation with clinical data and laboratory results is essential to avoid over-interpretation of incidental calcifications in older adults [14].

Semi-quantitative burden scores, though not universally adopted, can improve longitudinal consistency. Reporting whether calcification is confined to the globus pallidus or extends to the putamen, thalami, and dentate nuclei helps the geneticist and endocrinologist triage. Dual-energy CT and susceptibility-weighted MRI can aid differentiation from iron or other minerals in equivocal cases, but availability varies.

In secondary Fahr syndrome due to hypoparathyroidism, metabolic optimization is the cornerstone of care. Intravenous calcium is indicated for symptomatic hypocalcemia, followed by oral calcium and activated vitamin D (for example, calcitriol) with targets in the low-normal serum calcium range to minimize hypercalciuria. Across published case series and reports, correction of hypocalcemia and hyperphosphatemia leads to consistent improvement in neurological symptoms-particularly seizures, encephalopathy, tetany, and gait disturbance-usually within days to weeks of stabilizing the mineral axis. Movement and psychiatric features may improve more slowly but often respond to a combination of metabolic control and standard symptomatic therapies. In contrast, intracranial calcifications on CT almost always persist or change minimally, highlighting that “symptom improvement” refers to clinical and functional outcomes rather than radiologic reversal. Patients and families should be explicitly counseled about this dissociation to avoid unrealistic expectations while reinforcing the value of meticulous metabolic follow-up [13-15].

Management principles differ by etiology

For PFBC, no disease-modifying therapy exists; care is symptom-directed and multidisciplinary. Parkinsonian features may respond variably to dopaminergic therapy; dystonia can be approached with anticholinergics or botulinum toxin in focal forms; chorea may respond to VMAT-2 inhibitors or atypical antipsychotics; seizures are treated with standard antiseizure medications, avoiding agents that exacerbate dystonia when possible. Neuropsychiatric symptoms warrant the same evidence-based approaches used in primary psychiatric care, with attention to drug-symptom interactions (e.g., neuroleptics and parkinsonism). Rehabilitation (physiotherapy, occupational, and speech therapy) is often overlooked but essential to maintain function. In secondary Fahr syndrome due to hypoparathyroidism, metabolic optimization is the cornerstone: intravenous calcium for symptomatic hypocalcemia, followed by oral calcium and activated vitamin D (e.g., calcitriol). Therapeutic targets favor the low-normal serum calcium range to mitigate hypercalciuria; serum phosphate should be controlled and urinary calcium monitored. PTH analogues may be considered in selected chronic hypoparathyroidism under specialist supervision, but evidence for any effect on intracranial calcifications is limited. Crucially, patients and families should be counseled that symptomatic improvement is expected with biochemical correction, even if imaging appearances do not change.

For PFBC-related movement disorders, medication choice is individualized. Levodopa responsiveness is inconsistent; small case series suggest that dopamine agonists may help selected patients but can worsen impulse control disorders. For dystonia, anticholinergics may provide relief at the expense of cognitive side effects in older adults; focal forms respond well to botulinum toxin injections. Chorea can be mitigated with VMAT-2 inhibitors, mindful of depression risk. Seizure control generally follows standard practice; sodium-channel agents and levetiracetam are commonly used [3,16].

In chronic hypoparathyroidism, patient education about adherence, dietary calcium, and hydration is central to minimizing oscillations in serum calcium. Monitoring includes periodic serum electrolytes, urinary calcium/creatinine ratios, and renal ultrasound when hypercalciuria is suspected. Collaboration between endocrinology, neurology, and nephrology prevents therapeutic tunnel vision.

For nephrologists, management of BGC is anchored in brisk but safe correction of the mineral axis. Symptomatic hypocalcemia should be treated with intravenous calcium followed by maintenance oral calcium and active vitamin D, aiming for low-normal serum calcium levels to reduce the risk of hypercalciuria and soft-tissue calcification. Phosphate control should be optimized with dietary restriction and, when needed, non-calcium-based binders, particularly in patients with end-stage kidney disease (ESKD) in whom excess calcium load may aggravate vascular calcification. Symptomatic neurological therapy proceeds in parallel, using standard antiseizure medications for epilepsy and conventional agents for parkinsonism, dystonia, or chorea in collaboration with neurology. Dialysis prescriptions should be reviewed to avoid rapid mineral shifts and to ensure appropriate dialysate calcium and phosphate control. In the perioperative setting after thyroid or parathyroid surgery, clinicians should anticipate hungry-bone physiology and adjust calcium and calcitriol proactively to prevent neurological decompensation. Following kidney transplantation, renewed attention to calcium, phosphate, and PTH is required because hypoparathyroidism may re-emerge or become unmasked as renal function improves. Across these scenarios, patient education about adherence, diet, and hydration, together with regular biochemical monitoring, is essential to translate biochemical correction into sustained neurological benefit [1,17].

Special populations and scenarios

Intracranial arterial calcifications (ICAC) are highly prevalent in hemodialysis populations and appear to correlate with dialysis-specific risk factors, including higher phosphorus and inflammatory burden; higher ICAC scores have also been associated with increased short-term mortality. Although ICAC represents vascular rather than parenchymal calcification, it may serve as a pragmatic marker of systemic calcification propensity in advanced CKD. Contemporary reviews note that extravascular soft tissue calcifications in CKD, including brain involvement, are recognized but remain incompletely characterized. We therefore suggest that prominent ICAC on neuroimaging should prompt a focused, individualized review of phosphate balance, calcium exposure, and overall CKD-MBD and cardiovascular risk care, without implying a proven causal link to parenchymal BGC.

Transplant

Fluctuations in PTH and mineral metabolism pre-/post-transplant warrant vigilance; symptomatic PFBC is rare, but metabolic Fahr may flare with severe hypocalcemia.

Pregnancy

Coordinate obstetric, endocrine, and nephrology care; avoid profound hypocalcemia and hyperphosphatemia.

Saudi Arabia: published experience and opportunities

Published Saudi Arabia cases remain few and are primarily hypoparathyroidism-related (see Table 1). Reports include adult and pediatric presentations ranging from tetany and seizures to involuntary movements and encephalopathy. Imaging consistently demonstrates bilateral basal ganglia-predominant involvement with variable spread to dentate nuclei and subcortical white matter. Clinical improvement after calcium and vitamin D optimization is a recurring theme, while calcifications persist-mirroring international experience. The small number of published cases likely reflects under-recognition or under-reporting rather than true rarity; a national registry or multicenter case series would more accurately define burden, phenotypes, and outcomes.

**Table 1:** Illustrative Fahr/related (BGC) cases reported from Saudi Arabia CT: computed tomography; Ca: calcium; P: phosphate; PTH: parathyroid hormone; BGC: basal ganglia calcification; BG: basal ganglia; WM: white matter; KSMC: King Saud Medical City

Year	City/institution	Age/sex	Etiology/context	Presentation	Imaging	Outcome/notes	Source
2009	Hafr Al-Batin (King Khalid General Hospital)	Adult	Idiopathic hypoparathyroidism	Tetany ± seizures	Basal ganglia calcifications (CT)	Prompt Ca/P/PTH screening recommended	Basak [10]
2017	Taif (Armed Forces Hospital-Pediatrics)	11/M	Idiopathic hypoparathyroidism	Tetany; no focal deficits	BGC + extra-BGC	First Saudi pediatric report with extensive extra-BGC	Kamal et al. [11]
2020	Riyadh (KSMC/Sultan Military; Jeddah collaborators)	33/F	Hypoparathyroidism	Chvostek/Trousseau; involuntary movements	BG + subcortical WM + dentate	Improved after Ca/Vit D	Khan et al. [18]
2023	Qassim (Sulaiman Al-Rajhi University)	63/F	Post-thyroidectomy hypoparathyroidism	Recurrent seizures, encephalopathy	Widespread (BG, corona radiata, cerebellum)	Neurologic improvement with metabolic correction	Jihwaprani and Kumara [19]

Knowledge gaps and research agenda

Important knowledge gaps remain. First, the true prevalence of clinically significant BGC in CKD, ESKD, and post-transplant populations is unknown, as available evidence is limited to isolated case reports rather than cohort or retrospective studies. Second, there are no prospective data on neurological outcomes after optimization of CKD-MBD targets. Third, whether disturbances in mineral metabolism accelerate phenoconversion in PFBC heterozygotes remains untested. Finally, pragmatic care pathways are needed to reduce time-to-treatment while avoiding unnecessary genetic testing.

Global context and comparison

This review highlights a recurring practical theme: BGC sit at the intersection of neurology, endocrinology, and nephrology, and missed opportunities most often relate to failure to request a simple mineral panel when calcifications are first reported. Embedding a short checklist into radiology reports-commenting on distribution and symmetry and adding a one-line reminder to consider Ca/Phos/PTH/vitamin D testing when calcifications are unexpected or extensive-is a low-cost systems intervention rather than a rigid protocol. In routine practice, this kind of structured reminder does not replace clinical judgment and will not trigger mandatory testing in every patient, but it can shorten time-to-diagnosis in those with unrecognized hypoparathyroidism or advanced CKD-MBD by nudging clinicians to consider readily correctable metabolic causes early (Table 2).

**Table 2:** Global comparison-PFBC (genetic) versus secondary Fahr PFBC: primary familial brain calcification; Ca: calcium; P: phosphate; PTH: parathyroid hormone; GP: globus pallidus; WM: white matter

Feature	PFBC (genetic)	Secondary Fahr (e.g., hypoparathyroidism)
Mechanism/genes	Phosphate transport and neurovascular-unit biology; SLC20A2, PDGFRB, PDGFB, and XPR1 (dominant) and MYORG, JAM2, and CMPK2 (recessive) [3-6,14]	Disturbed Ca-P-PTH homeostasis; toxins/infections and other systemic disorders [3-6,14]
Clinical picture	Movement and neuropsychiatric features; seizures variable; many remain asymptomatic [3-6,20]	Hypocalcemic symptoms frequent; movement/psychiatric features possible
Typical age	Often 30-50 years with incomplete penetrance [4-6]	Any age (depends on cause)
Imaging hallmarks	Bilateral symmetric GP/putamen ± dentate/thalami/subcortical WM [3-6]	Radiologic overlap with PFBC; may be more widespread when metabolic disease is long-standing [1,15]
Management emphasis	No disease-modifying therapy; symptomatic and multidisciplinary care [3-6]	Treat the cause (calcium/active vitamin D; manage phosphate); symptoms often improve, though calcifications persist [15]

This narrative review highlights a recurring practical theme: BGC on CT is a pattern that can arise from several mechanisms; the clinical imperative is to separate actionable secondary causes from PFBC in a timely and proportionate manner. Our suggested pathway-non-contrast CT confirmation followed by a compact metabolic panel in all patients and targeted genetic testing when indicated-respects this imperative. In real-world settings, over- and under-investigation occur in equal measure. Some patients undergo extensive neuroimaging and infectious and autoimmune serology before basic calcium-phosphate-PTH testing is performed, delaying therapy; others, particularly older adults with incidental calcification, receive unnecessary genetic testing that is unlikely to change care. Embedding a short checklist into radiology reports (distribution, symmetry, and a reminder to check Ca/P/PTH/Vitamin D) may reduce this variability [1-4,7,8].

From a nosological standpoint, the genetic era has clarified the PFBC entity yet also exposed its heterogeneity. The overlap of movement, cognitive, and psychiatric phenotypes across genotypes suggests that downstream network effects-rather than the mere presence of mineral-shape symptomatology. This may explain the weak correlation between radiologic burden and clinical severity noted in observational cohorts. It also cautions against therapeutic nihilism: even when calcifications are extensive, many patients benefit from meticulous symptomatic therapy and rehabilitation, especially when combined with optimization of vascular risk factors and sleep.

Saudi case reports mirror international experience by emphasizing secondary, hypoparathyroidism-related disease with good functional recovery after metabolic correction. The low publication volume should not be misread as rarity; incidental calcification is frequently seen in emergency CT suites. A national registry seeded within neurology and endocrinology clinics would rapidly clarify patterns of referral, genetic yield, treatment responses, and patient-reported outcomes. In the interim, clinicians can adopt a standardized follow-up package: monitor biochemical indices every 3-6 months in secondary disease; review motor, cognitive, and psychiatric status annually in PFBC; and provide written education on symptom triggers and when to seek urgent care (e.g., tetany, new focal deficits, or breakthrough seizures) [1,3,15,20].

Future directions

Future directions include harmonized natural-history cohorts to define trajectories across genotypes, refined imaging quantification (e.g., automated CT-based calcification scores), and interventional studies that target phosphate handling or neurovascular-unit integrity. For secondary forms, pragmatic trials comparing strategies for calcium/phosphate targets, PTH analogue use, and structured rehabilitation could clarify how best to translate biochemical control into durable neurological gains. From a systems perspective, establishing national or regional registries-beginning with simple, standardized case report forms-would immediately enhance visibility of the disorder in Saudi Arabia and the Gulf.

A realistic near-term goal is consensus on minimal work-up and follow-up packages-laboratory intervals, imaging frequency, and patient-reported outcome measures-to standardize care and generate comparable data across centers. For research, multiomic profiling of PFBC brain tissue and patient-derived cellular models may clarify whether mineral deposition is an endpoint or an active driver of neurodegeneration, thereby identifying druggable nodes.

Limitations

This review is narrative and non-systematic; although we prioritized recent, peer-reviewed, and open-access sources, we did not apply PRISMA methods or undertake duplicate screening and data abstraction. The Saudi case list is illustrative rather than exhaustive and includes one conference abstract; nevertheless, the convergent clinical message (secondary, hypoparathyroidism-related cases improve clinically with metabolic correction) is robust and consistent with international data.

## Conclusions

In patients with BGC, nephrologists are uniquely positioned to shorten the road from incidental imaging to effective care. A compact metabolic panel applied early can rapidly identify potentially reversible mineral axis abnormalities and iatrogenic or postsurgical contexts, including inappropriately low PTH states that may occur after neck surgery or within the spectrum of adynamic bone disease in advanced CKD. While secondary hypoparathyroidism-related cases often show symptomatic improvement after metabolic correction, direct evidence that optimization of CKD-MBD targets improves neurological outcomes in CKD/ESKD is currently insufficient. Accordingly, our approach emphasizes contextual interpretation of PTH; correction of symptomatic hypocalcemia, hyperphosphatemia, and hypomagnesemia; and avoidance of iatrogenic extremes of calcium load and PTH suppression, alongside selective use of genetics for PFBC. Clearer care pathways and regional registries-particularly in dialysis and transplant cohorts-are needed to define prevalence, phenotypes, and treatment-responsive outcomes.

## References

[1] Saleem S, Aslam HM, Anwar M, Anwar S, Saleem M, Saleem A, Rehmani MA (2013). Fahr's syndrome: literature review of current evidence. Orphanet J Rare Dis.

[2] Manyam BV (2005). What is and what is not 'Fahr's disease'. Parkinsonism Relat Disord.

[3] Batla A, Tai XY, Schottlaender L, Erro R, Balint B, Bhatia KP (2017). Deconstructing Fahr's disease/syndrome of brain calcification in the era of new genes. Parkinsonism Relat Disord.

[4] Nicolas G, Pottier C, Charbonnier C (2013). Phenotypic spectrum of probable and genetically-confirmed idiopathic basal ganglia calcification. Brain.

[5] Balck A, Klein C, Westenberger A (2025). Primary familial brain calcification overview. GeneReviews® [Internet].

[6] Chen SY, Ho CJ, Lu YT, Lin CH, Lan MY, Tsai MH (2023). The genetics of primary familial brain calcification: a literature review. Int J Mol Sci.

[7] Donzuso G, Mostile G, Nicoletti A, Zappia M (2019). Basal ganglia calcifications (Fahr's syndrome): related conditions and clinical features. Neurol Sci.

[8] Saade C, Najem E, Asmar K, Salman R, El Achkar B, Naffaa L (2019). Intracranial calcifications on CT: an updated review. J Radiol Case Rep.

[9] de Brouwer EJ, Kockelkoren R, De Vis JB (2020). Prevalence and vascular risk factors of basal ganglia calcifications in patients at risk for cerebrovascular disease. J Neuroradiol.

[10] Basak RC (2009). A case report of basal ganglia calcification-a rare finding of hypoparathyroidism. Oman Med J.

[11] Kamal NM, Alghamdi HA, Halabi AA (2017). Idiopathic hypoparathyroidism with extensive intracranial calcification in children: first report from Saudi Arabia. Medicine (Baltimore).

[12] Yang D, Lu Y, Huang H (2025). Gene variants related to primary familial brain calcification: perspectives from bibliometrics and meta-analysis. eNeuro.

[13] Soares FB, Amorim FF, Santana AR (2013). Fahr’s syndrome due to hypoparathyroidism following thyroidectomy. J Med Cases.

[14] Bollerslev J, Rejnmark L, Marcocci C, Shoback DM, Sitges-Serra A, van Biesen W, Dekkers OM (2015). European Society of Endocrinology clinical guideline: treatment of chronic hypoparathyroidism in adults. Eur J Endocrinol.

[15] Lamessa A, Tesfaye K, Woyimo TG, Gebremichael EH (2024). First-time seizure revealing late-onset Fahr's disease: a case report and brief literature review. Front Hum Neurosci.

[16] Malik N, Pattan V, Nai Q, Yousif A (2014). Hypoparathyroidism, hypothyroidism and thrombocytopenia: rare constellation of Fahr’s syndrome. J Endocrinol Metab.

[17] Magrinelli F, Jesuthasan A, Bhatia KP, Batla A (2025). Basal ganglia calcification: 'Fahr's disease'. Pract Neurol.

[18] Khan S, Alhamdan AS, AlGhamdi A, AlShehri A, Douba N, AlAblani H, Husain AZ (2020). SAT-357 first case in Saudi Arabia revealing Fahr syndrome secondary to hypoparathyroidism: a case report. J Endocr Soc.

[19] Jihwaprani MC, Kumara EG (2023). Fahr’s syndrome secondary to hypoparathyroidism presenting with paralysis and recurrent seizures: a case report. Asian J Med Health.

[20] de Brouwer EJ, de Jong PA, De Jonghe A (2021). Histology and computed tomography of incidental calcifications in the human basal ganglia. Neuroradiology.

